# Advances in Non-Destructive Early Assessment of Fruit Ripeness towards Defining Optimal Time of Harvest and Yield Prediction—A Review

**DOI:** 10.3390/plants7010003

**Published:** 2018-01-10

**Authors:** Bo Li, Julien Lecourt, Gerard Bishop

**Affiliations:** NIAB EMR, East Malling, Kent ME19 6BJ, UK; bo.li@emr.ac.uk (B.L.); Julien.Lecourt@emr.ac.uk (J.L.)

**Keywords:** pre-harvest, ripeness, image analysis, machine learning, fruit phenotyping

## Abstract

Global food security for the increasing world population not only requires increased sustainable production of food but a significant reduction in pre- and post-harvest waste. The timing of when a fruit is harvested is critical for reducing waste along the supply chain and increasing fruit quality for consumers. The early in-field assessment of fruit ripeness and prediction of the harvest date and yield by non-destructive technologies have the potential to revolutionize farming practices and enable the consumer to eat the tastiest and freshest fruit possible. A variety of non-destructive techniques have been applied to estimate the ripeness or maturity but not all of them are applicable for in situ (field or glasshouse) assessment. This review focuses on the non-destructive methods which are promising for, or have already been applied to, the pre-harvest in-field measurements including colorimetry, visible imaging, spectroscopy and spectroscopic imaging. Machine learning and regression models used in assessing ripeness are also discussed.

## 1. Introduction

There are many ways in which the status of global food security can be improved for the world’s increasing population. Increased fruit production through adding to the area cropped is not sustainable, thus productivity per unit land area must be increased. Simultaneously there is a need to prevent waste, and for fruit production the timing of harvest is crucial to ensuring that production meets the commercial ripeness specifications. Over- or under-ripe fruits have a lower or even no retail value and represent significant income loss and a waste of resources. For the consumer, too early harvest reduces the taste and quality of fruits whilst a late harvest can lead to reduced shelf life, poor appearance, and “off” flavours and odours. The early in-field assessment of fruit ripeness and the prediction of both harvest date and yield will therefore greatly reduce the waste in the supply chain and thus help towards improving food security. 

The non-destructive on-plant assessment of fruit ripeness has received increasing interest as it provides several advantages compared with traditional destructive methods, such as high-throughput assessment, simultaneous multiple measurements and real-time decision making. The phenotypic changes during fruit ripening are complex and in most cases a green hard immature fruit becomes more colourful, softer, sweeter and aromatic. Numerous physical and chemical attributes that can be quantified during ripening include size, shape, texture, firmness, external colour, internal colour, concentration of chlorophyll, soluble solids content (SSC), starch, sugars, acids, oils, and internal ethylene concentration [1]. It is not realistic to simultaneously assess all the quality attributes in the field with non-destructive methods, and destructive laboratory measurements are time-consuming due to the large number of samples required to take account of the within-field variability [2]. Simple representative non-destructive measurements are thus required to assess the ripeness of a fruit. 

The measurement of fruit maturity in a non-destructive manner dates back more than a half century ago with the development of light transmittance techniques [3,4]. Since then, a variety of non-destructive techniques have been introduced including colorimetry [5], visible imaging [6], visible and near infrared (VNIR) spectroscopy [7], hyperspectral imaging [8], multispectral imaging [9], fluorescence imaging [10], acoustic impulse technique [11], Computed Tomography (CT) scan [12], Magnetic resonance imaging (MRI) [13], the acoustical vibration technique [14] and the electronic nose technique [15]. The first six techniques listed above will be considered in this review as they are the most likely to be used in portable devices to enable pre-harvest in-field assessment of fruit ripeness. These techniques have been applied to studies of a large number fruits, and external quality attributes have been measured and correlated with internal characteristics, as shown in Table 1. The non-destructive methods can produce a large amount of data with multiple variables, and thus multivariate analysis is utilized to identify key discriminatory variables that correlate with the ripening status of a fruit [16]. Such key discriminatory variables can be used in regression models, enabling an assessment of fruit quality and thus ripeness.

**Figure 1:**
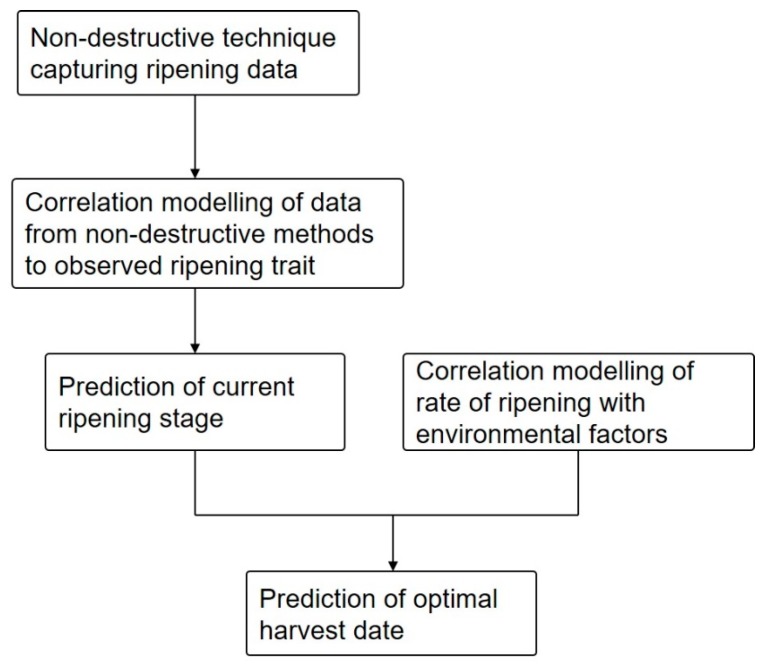
A scheme of the overall workflow for the prediction of the optimal harvest date.

Currently, the harvest time is mainly estimated by counting of days after flowering, subjective tasting, or visual assessment of fruit colour, texture or plant canopy structure [17,18]. All of these methods on their own or in combination are time consuming and not necessarily accurate. The quality attributes or the maturity indices derived from non-destructive techniques can, however, be modelled to predict the optimal time of harvest. Such predictions need to account for changes in environmental conditions and, being a primary factor affecting the rate of plant development [19], air temperature is a key parameter utilized in models predicting optimal harvest time [20]. An example of a typical approach in developing a work flow for predicting an optimal harvest date is given in Figure 1.

Here we review the non-destructive techniques for the fruit ripeness assessment and the modelling for the prediction of an optimal harvest time. These predictions are based on imaging and/or spectroscopic techniques that quantify the colour and/or spectral qualities of fruits that change due to their molecular composition during the ripening process.

## 2. Colour Measurement

The colour and appearance of a fruit is the initial quality assessment consumers use to judge the acceptability of a fruit. These criteria are related to physical and chemical changes occurring during fruit ripening [16,94]. In many fruits, colour change during ripening occurs due to chlorophyll degradation and the increase in the concentration of pigments such as carotenoids or polyphenols [95]. Several fruits have been studied for the relationship between maturity and colour including tomatoes [96], oranges [95], guavas [97], peaches [98], nectarines [44], mangos [46,99], blueberries [100], cherries [100] and pineapples [89]. To measure the changes in fruit colour, the two major methods are the use of colorimeters, and image capture and analysis.

### 2.1. Colorimeter

Colorimeters are traditional non-destructive instruments used extensively in the fruit industry to measure fruit colour [101]. They are more precise than human visual assessment and standardization using CIELAB colour space, which was introduced by Commission Internationale de l’Eclairage (CIE) in 1976, and provides unified measurements [102]. The three coordinates of CIELAB colour space, L*, a* and b*, represent the respective values of lightness and the green to red and blue to yellow ratios. CIELAB is close to human perception of colour due to the uniform distribution of colours, and all the colours that can be perceived by human eye can be located on the three coordinates [103]. Several colour indexes have been developed as the indicator of ripeness. In certain experiments only the a* parameter was correlated with colour change [97,102,104]. The b* value has only been reported to be positively correlated with ripeness in peaches [37]. Using more than one of the colour components improves the assessment of ripeness. The ripeness of tomatoes has been assessed using the ratio between a* and b*, which has shown high positive correlation with lycopene concentration [105,106,107] and which identifies significant differences for the six USDA ripening classes [56,101]. Equations based on a* and b* include determining the hue angle and chroma. The hue angle was found to be one of the best parameters to discriminate different ripeness stages for tomatoes, peaches and guavas [37,97,101,105,107]. The L* value was also incorporated to the colour models with a* and b* including for citrus [108] and tomatoes [102]. Portable colourimeters are now available commercially [46] and can be carried to the field. However, single fruit measurement is limited in its application to map the ripeness of fruits in the whole field. 

Data obtained with colorimeters has been successfully correlated with fruit ripeness by using multivariate analyses. These statistical tests allow the simultaneous model with multiple variables, for example multiple linear regression (MLR) has been used to accurately predict the maturity of mangos using a*, b* and their product as the variables [46]. Other regression methods were also tested in this study, including Partial Least Square (PLS) regression and Principal Component Regression (PCR), but the prediction performance were slightly worse than MLR in the study by Jha et al. [46].

### 2.2. Visible Imaging

Colorimeters are not able to obtain representative colour values due to the limited sampling area compared to the size of the fruit [109]. This limitation can be overcome by 2D colour imaging that converts photons reflected from fruit skin to electrical signals, which are then received by a camera with CCD (Charge-Coupled Device) or CMOS (Complementary Metal Oxide Semiconductor) sensors. Normally, the sensor receives the light and filters it to three channels, which are R (red), G (green) and B (blue), and the intensity values are always determined by fruit samples, illumination and the internal characteristics of the camera [110]. 

Similar to L*a*b* colour space, it is possible to analyze fruit ripeness in RGB colour space. Schouten et al. showed that the R component can be used to describe the progressive colour change of tomatoes at different stages of ripeness and that it correlates with changes in fruit firmness [59]. As ripening is a continuously changing process, the exact colour boundaries between different ripeness stages are difficult to determine and if used, arbitrary thresholds for each colour channel need to be provided. Fuzzy logic, a statistical analysis approach reviewed by Yuan and Klir [111], can overcome the need for discrete thresholds and has been applied to the ripeness assessment of mangos and apples [22,112]. Goel et al. used the difference between R and B values to enhance the classification of the different tomato ripeness stages, reaching 94.3% accuracy [113].

Other statistical methods utilized include unsupervised classification, such as K-means and Gustafson–Kessel algorithms, as reviewed in the studies of Hartigan et al. and Lesot et al. respectively [114,115]. These have successfully been applied to automatically separate bananas of different ripeness stages based on their RGB values [116]. Rather than only using the average RGB values, the histogram of each channel was used to find matches with predefined reference histograms for each ripeness group [116].

RGB values from images can be transformed to L*a*b* values, and a comparable performance was obtained with a colorimeter for the internal quality assessment of tomato fruits, including Brix and lycopene content [117]. A similar conclusion was drawn from studies in tomatoes [118], cherries [100] and bananas [94]. The RGB values are, however, device dependent and not a perceptually uniform space. Calibration is therefore crucial before the transformation of RGB values into L*a*b* space in order to produce parameters comparable with a colorimeter [103]. Statistical modelling approaches including quadratic and neural network models, as described in [119,120], were the best models to convert RGB values into L*a*b* space [110], and Taghadomi et al. adopted the neural network method and obtained a very strong correlation (R^2^ = 0.99) between actual L*a*b* and RGB values in cherries [100].

Other colour space values such as H, S and I (for hue, saturation and intensity) and H, S and V (hue, saturation and value), can be derived from RGB values and can better represent human visual perception [89]. Hue is defined as the similarity to the defined colours (red, green, blue and yellow) [121] and saturation is used to describe how colourful a stimulus is relative to its own brightness [122]. Fuzzy logic was successfully applied to group pineapples into three ripeness stages using values derived from H, S and I [89]. Ukirade et al. used H, S and V values as the input of the neural network model to classify tomatoes into four ripeness groups [123]. EI-Bendary et al. proposed a more sophisticated method for tomatoes, which used the colour histogram in HSV space and the colour moments (mean, standard deviation and skewness) which measure the colour distribution in an image as the colour features. Principal Component Analysis (PCA) was applied to extract the features for both Linear Discriminant Analysis (LDA) and Support Vector Machine (SVM) models with more than an 80% Correct Classification Rate (CCR) for five ripening stages [55]. Rather than only using one colour space, colour components from two colour spaces can be combined, such as in the study by Li et al., which used R, B and H from outdoor colour images of blueberries in combination with the K-Nearest Neighbour (KNN), which classifies an object based on a majority vote among its neighbours, [124] to achieve more than an 85% CCR when separating fruit into four ripening stages [125].

Compared with a colorimeter, colour information can be obtained rapidly from larger area in a 2D image due to the high spatial resolution. The equipment can also be easily attached to moving platform such as tractor, robot or drone for the rapid collection of multiple data collection measurements. There are however challenges that need to be overcome for 2D imaging including the difficulty of segmentation of fruits from the background, device dependent RGB values, and the requirement of homogeneous illumination in the field. 

## 3. Visible and Infrared Spectroscopy

**Figure 2:**
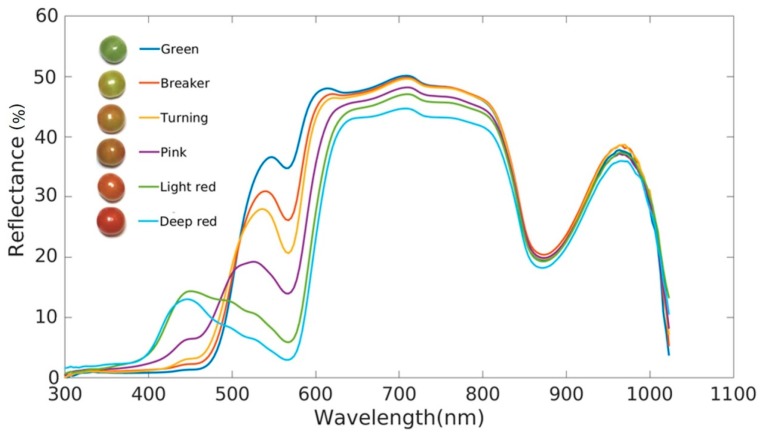
Typical progressive change of reflectance spectra at different ripening stages of tomato.

When light hits the surface of a fruit it can be absorbed, scattered or re-emitted. The amount of each of these is determined by the physical properties and chemical constituents and thus ripeness of a fruit. Visible and Near InfraRed (VNIR) reflectance spectroscopy measures reflected light between 380 nm and 2500 nm, which is largely dependent on the light absorption by fruit sample and relates to almost all the major organic compounds. An example of the changes in spectra during ripening is given in Figure 2, which shows marked changes in the values between 400–700 nm and again at longer wavelengths. VNIR spectroscopy has been widely applied as a non-destructive and fast measurement method for multiple quality attributes [126]. More importantly, a portable device has been developed and used in the field [127]. The recorded spectra can be analyzed and related to different ripeness stages by using spectral indices. The whole wavelength scan, or values at key selected wavelengths, are used in regression models to correlate with specific fruit qualities that are associated with fruit ripeness.

### 3.1. Spectral Indices

Spectral indices normally combine the surface reflectance at two or more wavelengths in order to indicate the relative abundance of a feature of interest. (It is difficult to use only one wavelength as an index for in-field assessment because the values can be highly affected by the sensor, environmental illumination and particle size [128].) A number of spectral indices have been calculated to describe the progressive change of peel pigment concentration during ripening. Ruiz-Altisent et al. found that the reflectance at 450 nm and 680 nm were both associated with peach firmness, but when using the reflectance values from only two wavelengths, the linear correlation factor was low (R^2^ < 0.6) [129]. The index of absorption (*I_AD_*) introduced by Ziosi et al. is a robust spectral index obtained by calculating the difference between the absorption at two wavelengths around the chlorophyll-a peak (670 nm and 720 nm) [39]. The *I_AD_* range was found to be similar across different growing seasons and a good correlation with ripeness was found for peaches [98,130], apricots [131], nectarines [132,133] and plums [92]. Merzlyak et al. identified several spectral indices for the quantification of internal quality attributes of apples related to their ripeness with high correlation (R^2^ > 0.8) [24] and Zude et al. also developed three indices, including the ratio between the transmission at 698 nm and 760 nm, NDVI and red-edge vegetation stress index (RVSI). Even though all these indices showed good correlations with the colour change of apple peels, the correlation appears to be cultivar dependent and had lower accuracy for the cultivar of ‘Jonagold’ than “Elstar” [23]. The same phenomenon was also noticed in the study by Shinya et al., where the correlation between firmness and *I_AD_* could be extremely different among three cultivars of peaches [98].

### 3.2. Full or Selected Wavelengths

Spectral indices can describe the change of peel pigment concentration during the ripening process and provide comparable values with the colorimetric method [37], but peel colour is not always the only criteria for ripening assessment. The correlation between internal quality attributes related to ripening, such as firmness and SSC, were investigated with full or selected wavelengths from the VNIR spectra.

For the full wavelengths, PLS is the most used regression model to predict fruit quality. The prediction is achieved by extracting a set of orthogonal factors from the predictors, called latent variables, which have the best predictive power [134]. Another common regression model is the Principal Component Regression (PCR), which uses Multiple Linear Regression (MLR) to correlate with the principal components scores extracted from the predictors [135], and which has been applied in some studies for fruit quality assessment [67,135,136]. Compared with PLS, PCR showed the drawback that the principal components were obtained without considering the dependent variables.

The variability of physical sample properties and/or the performance of the hardware can result in undesired results including light scattering, path length variations and random noise generated in the extracted spectra. These factors reduce the accuracy and robustness of the prediction models [137]. In order to improve the data analysis, a number of studies have applied different pre-processing techniques to the spectra obtained before modelling [136].

Savitzky–Golay (SG) is the most frequently used digital data smoothing filter [42,49,65,91,138,139], which applies the Linear Least Squares method to fit low-degree polynomial data [140]. However, SG has contrasting effects on the performance of multivariate statistical models [141]. For example, Jha et al. compared different pre-processing techniques and found that smoothing did not produce any improvement in comparison with other techniques for the assessment of the firmness in mangos [49]. But Herrera et al. showed that SG filters using a second-order polynomial performed better than other scattering correction methods for the prediction of wine grape Brix [137]. Standard Normal Variate (SNV) [72,78,142,143] and Multiple Scattering Correction (MSC) [42,67,137,139,144,145] are the two most frequently used techniques for scattering correction. MSC is used to eliminate the nonlinear scattering due to the non-uniform travel distance of light by linearizing each spectrum to a reference spectrum, which is the always the mean spectrum [67]. Previous research has shown the similarity between SNV and MSC, for example Ma et al. confirmed that the correlation coefficients were the same when assessing the sugar content of peaches using PLS models with SNV and MSC [146]. SNV can, however, be applied to an individual spectrum without requiring a reference [147]. In some studies, SNV was performed with de-trending, which was used to correct the baseline shift of spectra [72,143].

Generating derivatives of spectra are useful pre-processing techniques to enhance subtle differences and reduce the effect of specular reflection [79,137]. Guo et al. found that the PLS regression model performed better with the first derivative of the spectra than SNV, MSC, and the second derivative for predicting the SSC in strawberries [139]. A similar conclusion was drawn for the Total Soluble Solid (TSS) content prediction of strawberries [73]. The second derivative has also been used in the prediction of chlorophyll content of apples [23], the SSC of kiwifruit, strawberries, cherries, and peaches [71,79,148,149], and the firmness of apricots [78]. Carlini et al. compared the second derivative, MSC and SNV methods, and found that the second derivative showed the best performance for the prediction of SSC in cherries [71]. Interestingly, pre-processing techniques are not always beneficial to the spectral analysis. Clément et al. applied all the above-mentioned pre-processing techniques to the prediction of tomato ripeness, but it was found that none of them showed improvements on a PLS model due to the low levels of noise [61]. Likewise, Jaiswal et al. also found that the best predictions of TSS and DM (Dry Matter) content of bananas with PLS model was obtained with no pre-processing to the spectra [52].

In some studies, only a small number of selected wavelengths were used to reduce the multicollinearity among variables and to be modelled by the Multiple Linear Regression (MLR) model. The key wavelengths can be identified manually or automatically. The manual selection of key wavelengths has been used for the prediction of Brix in mangos [150] and SSC for grapes, limes and star fruit [84]. Guthrie et al. calculated the correlation coefficients between the second derivative of spectra and Brix values as the criteria of wavelength selection for the MLR model. This method provided better predictions than when using the first derivative [90] and had previously been used in the prediction peach Brix levels [151].

Automated wavelength selection methods, such as stepwise wavelength selection, have also been used to aid the predictive power of models. The first wavelength selected is that with the highest correlation to the depended variable. Additional wavelengths are added, one by one, in order to strengthen the correlation until none of the remaining wavelengths are significant. This method was used for the SSC prediction of peaches [152], melons and pineapples [153]. The Genetic Algorithm (GA), which uses natural selection and random mutations based on prediction accuracy, is another efficient automated method for identifying key wavelengths and was successfully applied to the SSC prediction of apples [154].

A comparison of the performance of PLS and MLR for the assessment of the maturity of mangos indicated that when using MLR, a poorer correlation of data was observed and led to overfitting, as seen by the large gap between the correlation coefficients of calibration and validation models [155]. A similar phenomenon was also observed for the prediction of TSS and DM for bananas [52]. However, if the key wavelengths that were selected represented most of the variance of the whole spectra but low collinearity, MLR can show better performance than PLS, such as in the firmness prediction of mangos [50]. These two methods of modelling were also compared for the prediction of Brix values in mangos and, interestingly, both correlation coefficients were high and comparable [150]. Consequently, it is unclear which model can provide a better prediction, as the performance of MLR is largely dependent on the wavelength selection.

The correlation has always been higher for the prediction of SSC than of the firmness of fruit by using spectroscopic methods. Park et al. showed that the prediction of firmness was more complicated than SSC as it was not determined by a single analyte or a limited group of related chemicals [156]. For both SSC and firmness prediction, the performance of the model is always cultivar dependent, and the calibration model trained by using mixed cultivars produces a lower correlation than when using results from an individual cultivar. A limited number of studies were focused on the assessment in-field, but compared with indoor measurement, the prediction is less accurate [27,45,48].

Spectroscopic methods utilize longer wavelengths than colorimeter and visible imaging, but similar to colorimeter, they are not likely to be applied as high-throughput ripening assessment tools due to the low spatial resolution. The accuracy of the internal quality measurement is influenced by sample temperature, which needs to be compensated for by an extra calibration model [48]. Spectroscopy has been used in assessing the quality of a large variety of fruits, and portable commercial spectrometers have been developed [48,78,127,157], but most of the studies have focused on the indoor, post-harvest assessment of fruit maturity. Inconsistent performances were observed for the models developed by spectra taken indoor and on-tree. Predicting apple firmness and SSC both on the tree and during storage showed that the on-tree PLS model had the best correlation coefficients for both firmness and SSC [27,158]. However, for nectarines, the on-tree model performed worse than the post-harvest one [45]. Consequently, for the on-tree ripeness assessment, it is necessary to build the prediction model with spectra taken in-field and understand the effect of environmental factors on the quality of spectra.

## 4. Fluorescence Sensor

Fruit degreening (i.e., the loss of chlorophyll) is an effective indicator of fruit ripening, and thus measuring chlorophyll content using a fluorimetric sensor can be correlated to fruit ripeness traits [40]. 

One chlorophyll fluorimetric method measures the photochemical and non-photochemical processes with the illumination of actinic light [159]. A Pulse-Amplitude-Modulation (PAM) based fluorometer has been developed commercially, which uses visible light at the blue region as the excitation and measures the minimum (*F*_0_) and maximum (*F_m_*) emitted fluorescence. The maximum quantum yield (*F_m_ − F*_0_)/*F_m_* was calculated and this parameter was found to be negatively correlated with the ripening stage of apples [160] and papaya fruit [161]. This chlorophyll fluorimetric method is popular in the laboratory, but difficult to apply in-field as the samples need to be dark-adapted. For example, the papaya samples in the study of Urbano et al. were dark-adapted for 30 min with a dark towel [161]. Bodria et al. designed a fluorescence imaging system which measured the light emission at 690–740 nm with the excitation light in UV-blue and red regions, and a good correlation was achieved between the quality parameters, including firmness and SSC of fresh apples, and detected fluorescence, even though the hue value of the skin colour showed little change. However, the correlation was lower for peaches and nectarines [40]. The fruit samples measured by this fluorescence imaging system were not dark adapted, but the equipment has only been designed for the laboratory use. 

In order to reduce the influence of environmental factors on the absolute fluorescence intensity at a single band, which limits in-field application [162], more studies were focused on the understanding of the fluorescence ratios using various light sources of defined wavelengths. This has led to the development of a handheld, multi-parametric fluorescence sensor: Multiplex^®^ (Force-A, Orsay, France), with four LED light sources and three synchronized fluorescence detectors [10]. The most common informative indices utilized fluorescence from anthocyanins (ANTH), flavonols (FLAV), and chlorophyll (CHL) to indicate fruit ripeness. In the study of Betemps et al., CHL showed a positive correlation with the firmness of apples and the authors also obtained a good negative correlation between FR_RED and SSC [29]. The in-field assessment of CHL was also successfully applied to grapes, with a high correlation with TSS, and the combination of CHL and ANTH can be used as a robust decision tool to predict harvest time [86]. For tomatoes, all the indices were found to correlate well with the time-shift in the tomato ripening process in the study of Hoffmann et al. [62]. The blue to red fluorescence ratio (B_UV/RF_UV) was measured as an effective parameter for the assessment of the ripeness of oil palm with rough skin, and when combined with the Classification and Regression Tree (C&RT) method resulted in an overall correct classification rate of 90% for three different ripeness stages [163]. Current studies of fluorescence sensors are focused only on the analysis of specific parts of a fruit, which limits their potential use for high throughput measurements in the field. 

## 5. Spectral Imaging

### 5.1. Hyperspectral Imaging (HSI)

Hyperspectral imaging (HSI) has emerged as a powerful tool for the inspection of fruit quality. HSI generates a three-dimension imaging cube with images at a range of continuous wavelengths. A single spectrum can be extracted from each individual pixel representing the absorption properties and the textural information of fruit samples [164]. Similar with traditional visible imaging and spectroscopic methods, HSI is non-destructive and requires little sample preparation, but it is advantageous in that it can record both spatial and spectral information simultaneously [165]. For the assessment of fruit quality, two types of wavelength dispersion devices are normally used i.e., line scanning and area scanning coupled with an imaging sensor for the HSI image acquisition. A line scanning device has the imaging spectrograph dispersing the incident light into different wavelengths instantaneously between the visible and the near infrared wavelength range (380–1700 nm). Line scanning HSI cameras scan the samples continuously in one direction, so they can be attached to moving platforms such as tractors [166], robots [167] and unmanned aerial vehicles (UAV) [168].

The hyperspectral image can be handled in two different ways (1) light scattering analysis and (2) spectral analysis. Modified Lorentzian Distribution (MLD), which correlates the data obtained with a predefined distribution curve by using a distribution function, can be used to describe the scattering profile and the fitting parameters that were used as the variables for the stepwise MLR model [30]. The results of this study suggest that spectral scattering from all wavelengths or selected wavelengths can provide more accurate predictions of apple firmness than using the secondary properties such as spectral absorption [30]. Similar methods were also employed for the prediction of peach firmness, but with MLR models, different results were obtained when using two different cultivars [41]. Mendoza et al. combined both the spectral and image analysis techniques on scattering images, including discrete and continuous wavelet transformation decomposition, first order statistics, Fourier analysis, co-occurrence matrix, and Variogram analysis, but little improvement on the prediction of firmness and SSC of apples was found, with the performance of the PLS model being cultivar dependent [31]. Wang et al. used two different feature selection methods which were Uninformative Variable Elimination (UVE) [169] and Supervised Affinity Propagation (SAP) [170]. The output of two PLS models with two feature selection methods were combined as the input into an Artificial Neural Network (ANN) model that gave a correlation coefficient of 0.83 [171]. The scattering profile was also used for the prediction of firmness of peaches [41].

The average spectra of the region of interest (ROI) have also been modelled for the assessment of fruit quality. The average spectra from whole wavelength scan (400–1000 nm) were used with a PLS model to predict the SSC of grapes. The correlation coefficients were similar for both white and red grapes [88]. The same method has also been used to predict the TSS of strawberries [75], and the firmness and SSC of blueberries [172]. Key wavelengths have also been selected, with different feature selection techniques, before modelling in order to reduce the redundancy of the whole spectral dataset. One of widely used feature selection criteria is based on beta coefficients derived from PLS models. The PLS models measured how great effect an independent variable has on the dependent variable. A comparison of the performance of different MLR models with wavelength selection based on beta coefficients and PLS models with full spectra as input, showed that the final outcomes were similar for the internal quality measurement of strawberries [75]. The same feature selection methods were also used by Rajkumar et al. to predict the firmness and TSS of bananas by MLR model, and a good correlation was achieved for both quality attributes with correlation coefficients of 0.91 and 0.85, respectively [54]. Another key wavelength selection method which can solve the collinearity problem is the Successive Projection Algorithm (SPA), which iteratively adds wavelengths one by one until a specific number of wavelengths is achieved with a minimum redundant information content [173]. This method has been used to select the feature wavelengths for input to the PLS model and a high correlation (R^2^ = 0.92) was found for predicting persimmon firmness [83].

Overall HSI is a promising technique for fruit ripeness assessment. In-field application of this technique will need to overcome the challenges of handling the large data output and the calibration of variable light levels whilst in the crop. 

### 5.2. Multispectral Imaging (MSI)

Multispectral imaging is a form of HSI that collects data at specific wavelengths instead of scanning the whole wavelength range. This can be accomplished using a frame scanning imaging system with Liquid Crystal Tunable Filter (LCTF) coupled with CCD or CMOS sensor. Lu et al. used five wavelengths, based on previous studies, to correlate their scattering profiles with the firmness and SSC of apples using an ANN model. They obtained a reasonably high correlation for both quality attributes, which were R^2^ = 0.87 and 0.77, respectively [174]. Another lower-cost MSI system uses a rotating filter wheel containing a few bandpass filters instead of LCTF, but the tuning speed is lower than LCTF. This device has been used to predict the firmness and SSC of peaches with the best combination of four wavelengths, and high correlation coefficients were achieved; 0.94 and 0.97, respectively [76]. The prediction of firmness was higher than the prediction by HSI [41]. Similarly, Liu et al. used MSI with 19 wavelengths to predict the firmness and TSS of strawberries. PLS, SVM and ANN were compared with the best correlation coefficients of 0.94 and 0.83, respectively [74], which were comparable with HSI for prediction of TSS of strawberries [75].

Among the techniques described above, MSI is the most promising for in-field measurement as it can record high resolution images at selected key wavelengths for the prediction of specific quality attributes. Compared with HSI, MSI can be lower cost and easier to convert into portable devices, and the output imaging dataset will be smaller. A portable MSI device with four narrow-band light sources and four reflectance sensors of different wavelengths at 570, 670, 750 and 870 nm has been developed [130]. This device was used to classify oil palm into different stages of ripeness using quadratic discriminant analysis and discriminant analysis with Mahalanobis distance classifiers with a correct classification rate of >85% being achieved [175].

## 6. Prediction of Optimal Harvest Date

Non-destructive methods are very promising for the ripeness assessment in-field, but the most critical question is to how to link such assessment to predict yield and the optimal harvest date [18]. This is highly challenging and made complicated by ripeness variability within and between plants.

Yang et al. recorded the HSI spectra of tomatoes at different growing stages, and the PLS model was applied to predict the growing stage with the best correlation coefficient being 0.89. It was also found that the key wavelengths were in the visible and infrared regions (400–2100 nm) [176]. A Similar method was employed to predict the number of days until the commercial harvest of apples. The calibration model was built with eight cultivars and a good correlation (R^2^ = 0.93) was found for the spectral range between 380 nm and 2000 nm [177]. Environmental factors such as temperature, light levels, humidity, etc. significantly influence the development of crop fruit, and it is essential to incorporate important environmental factor predictions for the determination of the optimal harvest date [178]. Several crop models have been developed since the 1960s by Loomis et al. [179] with the input of environmental factors both for in-field and greenhouse prediction. Such models are difficult to use due to the number of input variables [180,181,182,183,184,185]. Qiu et al. investigated the dominant environmental factors in greenhouses for tomato growth and it was found that temperature, humidity and Photosynthesis Active Radiation (PAR) show a positive or negative correlation to crop growth [185]. The influence of temperature has also been reported in a number studies such as for tomatoes [178], grapes [186], apples [187], mangos [188], blueberries [18], apricots [189] and strawberries [190]. Shewfelt et al. studied the colour change at different constant temperatures, but the variation of the temperature within each day was not considered [96]. Muñoz et al. developed a time series regression model for the prediction of the harvest date of blueberries, and the minimum and maximum of daily temperatures from the weather forecast for two weeks ahead were used as input for the model [18]. This method was closer to a real application and potentially could be applied with non-destructive techniques which could determine the current ripening stage. Environmental temperature affects the fruit growth, and it was also found that the fruit temperature also influenced the near infrared reflectance spectrum in a non-linear way [146]. Kawano et al. compensated for the surface temperature effect by developing a combined MLR model, which covered a variation of temperatures ranging between 21 and 31 °C [191]. Peirs et al. compared a global calibration model that covers a wide temperature range and calibration models for each temperature range. Both methods performed well for the SSC prediction of apples, but for the practical purpose, the global calibration model was preferred [26].

## 7. Conclusions

Although now slighted outdated, the key study by the Food and Agricultural Organisation (FAO) indicated that global food waste and losses in 2009 was estimated to be one-third (by weight) of all food produced in the world [192]. The prevention of such losses and waste is therefore a major driver to improve global food security. Here we have reviewed a range of non-destructive techniques and the data modelling methods for the assessment of fruit ripening and the prediction of the optimal harvest date. Knowing when to pick will not only depend on the fruit—to ensure the optimal taste, quality and postharvest performance—but also on the local circumstances including weather, the supply chain and markets. Having an affordable, portable device to inform this decision is crucial and the non-destructive techniques discussed above have all been developed into such devices, which will help to reduce waste.

Such devices include colorimeters that can record accurate colour information, but only for individual fruit. 2D imaging overcomes this limit as it images larger areas and can be fitted on a moving platform. However, 2D imaging can only obtain colour information, which is not adequate as an indicator of ripeness for all fruits. Similar to colorimeter and 2D imaging, fluorescence detects the colour change, especially the change of content of chlorophyll. Spectroscopy in visible and NIR regions correlates with both colour and internal quality attributes, which can provide a better prediction of fruit ripeness. Hyperspectral imaging has the advantage of both spectroscopy and 2D imaging, and can be integrated with a moving platform. However, due to the high expense and large dataset generation, it is still mainly used in the lab. Multispectral imaging has the potential to overcome the limitation of hyperspectral imaging but the reliability of the measurement in-field needs to be further investigated. Miniaturization and computational capacity will be major technical hurdles for ensuring that the devices can be used in the field and provide a real-time assessment of fruit quality and ripeness. Simultaneous to the development of the hardware needed for the data modelling techniques is important so that these solutions can utilize not only data from the controlled laboratory conditions, but also from the more challenging field conditions with a changeable environment. 

## Figures and Tables

**Table 1:** Overview of the non-destructive methods for the assessment of fruit ripening and their correlation with internal characteristics. Abbreviations: SSC (Soluble solid content), DM (Dry matter), MC (Moisture content), TTA (Titratable acidity), TSS (Total soluble solid) and WC (Water content).

	Colorimetry	Visible Imaging	Spectroscopy	Fluorescence	Hyperspectral Imaging	Multispectral Imaging
Apple	Colour [21]	Colour [22]	Chlorophyll [23], anthocyanins [24], carotenoids [24], flavonols [25], SSC [26], firmness [27]	Chlorophyll [28], anthocyanins [29], flavonols [29], firmness [29], SSC [29]	Firmness [30], SSC [31]	Firmness [32], SSC [33]
Pear			Firmness [34], SSC [35]		SSC [36]	
Peach	Colour [37]		Firmness [38], chlorophyll [39], colour [37]	Firmness [40]	Firmness [41]	Firmness [9], SSC
Avocado			MC [42], DM [42]		DM [43]	
Nectarine	Colour [44]		SSC [45], firmness [45]	Firmness [40]		
Mango	Colour [46]		DM [47], starch [48], SSC [47], colour [49], firmness [50]		Firmness [8], SSC [8], WC [8]	
Banana	Colour [51]	Colour [51]	TSS [52], Chlorophyll [53]		Firmness [54], TSS [54]	
Tomato	Colour [55], firmness [56], TSS [57]	Colour [58], firmness [59]	Lycopene [60], SSC [61]	Chlorophyll [62]		Phenolic [63], lycopene [63]
Melon			SSC [64]			
Mandarin			TTA [65], SSC [66], firmness [67], DM [68]			
Cherry	Colour [69]		Firmness [70], SSC [71]			
Strawberry			Colour [72], TSS [73], Firmness [72], TTA [73]		Firmness [74], TSS [75], TTA [75]	SSC [76], firmness [77]
Apricot			SSC [78], firmness [78], TTA [78]			
Kiwifruit			TSS [79], SSC [79], firmness [80], DM [81], Starch content [79]			
Persimmon			SSC [82]		Firmness [83]	
Grape			SSC [84], TTA [84], anthocyanin [85]	Chlorophyll [86], anthocyanin [86], TSS [86], flavonols [87]	SSC [88], TTA [88]	
Pineapple		Colour [89]	DM [90], SSC [91]			
plum			Firmness [92], SSC [93], colour [92]			
